# Efficacy, safety, and pharmacokinetics of rifaximin in pediatric patients with hepatic encephalopathy in Japan: An open-label study

**DOI:** 10.1097/HC9.0000000000001002

**Published:** 2026-07-15

**Authors:** Hideaki Okajima, Masashi Tagawa, Masaki Fujisawa, Kaoru Ueda

**Affiliations:** 1 Department of Pediatric Surgery, Kanazawa Medical University, Ishikawa, Japan; 2 ASKA Pharmaceutical Co., Ltd., Tokyo, Japan

**Keywords:** adverse effects, child, gut microbiota, hyperammonemia, mental aspects, portosystemic encephalopathy, rifamycin

## Abstract

**Background:**

To evaluate the efficacy, safety, and pharmacokinetics of rifaximin in children with hepatic encephalopathy (HE).

**Methods:**

We conducted a multicenter, open-label, phase II/III study, comprising a 12-week treatment period, in children aged <18 years with hyperammonemia (≥66 μg/dL) and grade I or II HE or suspected HE, and compared the results with those previously obtained in adults. Patients were treated with 400 mg rifaximin 3 times daily, as for adults. The primary endpoint was the blood ammonia concentration, and other endpoints were changes in mental state and number connection test (NCT)-A and -B results from baseline. The safety endpoints were adverse events and fecal microbial composition. The pharmacokinetics of rifaximin were also assessed.

**Results:**

Of 21 patients (median age 11.0 years old, Q1–Q3 [8.0–14.0]), 18 completed the study, with a median [Q1–Q3] treatment duration of 84.0 [83.0–85.0] days. The most common underlying disease was a congenital portosystemic shunt. Thirteen patients were suspected of having HE, and 8 were diagnosed with coma grade I. The serum ammonia concentration remained lower than that at baseline during treatment with rifaximin, although the difference did not reach statistical significance. There were significant reductions from baseline in the mental state associated with HE and NCT-B results at all time points. No new clinically relevant safety issues or marked changes in the composition of the gut microbiota were identified.

**Conclusion:**

This is the first pediatric clinical study to demonstrate the efficacy, safety, and pharmacokinetics of rifaximin in pediatric patients with HE. The efficacy signals were driven primarily by clinical and neurocognitive measures rather than biochemical (ammonia) changes. The safety and pharmacokinetics of rifaximin were similar to those for adult patients.

## INTRODUCTION

Hepatic encephalopathy (HE) is characterized by neuropsychiatric impairment, which is known to be associated with high blood ammonia concentrations.^1^^–^^3^ It is thought that the causes of HE include cerebral edema and altered neurotransmission caused by ammonia entering the brain and abnormal amino acid metabolism induced by hyperammonemia.

Because hyperammonemia appears to be responsible for the development of the clinical manifestations of HE, the control of the circulating ammonia is an objective of treatment, in addition to the management of underlying liver disease.^4^ Rifaximin (RIFXIMA) is a poorly absorbed rifamycin antibiotic drug that inhibits ammonia production in the gastrointestinal tract. Rifaximin was first approved in Italy in 1985 and has subsequently been marketed in 76 countries and regions.^5^

In Japan, rifaximin was approved for the indication of the “improvement of hyperammonemia in HE” for adults in 2016. However, no drugs have been marketed for the treatment of hyperammonemia in pediatric HE. There are differences in the underlying hepatic diseases present in children and adults with HE. For example, HE in adults is principally caused by chronic liver disorders such as viral hepatitis and alcoholic liver disease, whereas HE in children is typically caused by congenital diseases.^3^^,^^6^ However, considering the critical role played by hyperammonemia in the development of the neuropsychiatric impairment, rifaximin would be expected to be an effective treatment for HE in pediatric patients, as it is in adults.

To date, no clinical trials have been performed either in Japan or elsewhere, and there have only been a few case reports regarding pediatric HE, none of which have been performed in Japan. Therefore, the objective of the present study was to evaluate the efficacy, safety, and pharmacokinetics of rifaximin when administered to pediatric patients with HE for the treatment of hyperammonemia in Japan.

## METHODS

### Study design

We performed a multicenter, open-label, phase II/III study to evaluate the efficacy, safety, and pharmacokinetics of rifaximin in pediatric patients with HE for the treatment of hyperammonemia. The study was designed to mirror the previous phase II/III and phase III studies performed in adult patients with HE,^5^ such that the outcomes in the children could be compared with those in adults. In brief, the study consisted of screening, 12 weeks of treatment, and subsequent follow-up on day 85. The children were treated with rifaximin using the same dose regimen as for adults;^5^ 2 rifaximin 200 mg tablets (400 mg) administered orally 3 times daily after meals, at the same time of day whenever possible until day 84, or until an investigator decided to withdraw the patient from the study. The safety of rifaximin, which is poorly absorbed, has been established in adults at 400 mg 3 times daily.^5^ This study was designed to compare the safety and efficacy of rifaximin in pediatric patients with the previous results of safety and efficacy in adults. Therefore, pediatric patients were treated at the same dosage as adults in this study. Furthermore, to ensure the safety of pediatric patients, this study was designed to enroll pediatric patients and administer rifaximin in a stepwise manner under the supervision of the Safety Evaluation Committee because experience with the use of rifaximin in patients aged <12 years is limited.

Patients who were determined to be unable to swallow tablets were not enrolled in this study, and rifaximin was administered orally to all the enrolled patients. No modification in the administration of rifaximin, such as crushing or suspending tablets in fluid, was reported. No control or placebo group was included because there is no approved drug for the treatment of hyperammonemia in children with HE in Japan, and therefore, the treatment of patients with a placebo was deemed to be ethically inappropriate. Concomitant treatment with antibacterials (except for external use) or amino acid injections for hepatic failure, intended to ameliorate HE, was prohibited during the study.

If a patient developed a moderate or severe adverse drug reaction (ADR) related to the gastrointestinal tract (eg, diarrhea, constipation, abdominal distension, sickness, vomiting, or abdominal pain), the dose of rifaximin was reduced to 1 tablet (200 mg) 3 times daily. If the ADR is then resolved, the dose could be increased to its previous level.

If any of the following events occurred during the study, the patient was withdrawn: consent withdrawn by the patient or their legal representative, development of ≥coma grade III, withdrawal of the patient by an investigator because of an adverse event (AE) or protocol violation, pregnancy, loss to follow-up, death, or scheduled treatment (eg, liver transplantation) performed after day 15.

To ensure the safety of the children, rifaximin was administered in a stepwise manner because of limited experience with usage in patients aged <12 years. For patients aged 5–11 years, the first patient was administered rifaximin in step 1. Then, after the Data and Safety Monitoring Board found no issues with the safety data during the first 2 weeks of treatment, the second patient was started on rifaximin in step 2. After reviewing the data for the second patient, the third patient, aged 5–11 years, and the first patient, aged <5 years, were administered rifaximin in step 3. The second and third patients aged <5 years were then administered rifaximin in the same manner as in steps 1 and 2, respectively (steps 4 and 5). Patients aged >12 years were administered rifaximin without taking this extra precaution.

### Ethics statement

The study, protocol, and informed consent form were approved by the institutional review board of Kanazawa Medical University Hospital on 25 November, 2019 or the independent ethics committee at each study site. The study was conducted in accordance with the ethical principles derived from the Declaration of Helsinki; the Pharmaceuticals, Medical Devices, and Other Therapeutic Products Act; the Ministerial Ordinance on Good Clinical Practice; and other related laws and regulations. This study was reported in accordance with the STROBE (Strengthening the Reporting of Observational Studies in Epidemiology) guidelines. Written consent was obtained from the legal representative (parents or legal guardian) before participating in the study. Trial registration: Japan Registry of Clinical Trials (JRCT ID, jRCT2080224978); https://jrct.mhlw.go.jp/en-latest-detail/jRCT2080224978.

### Patients

Patients who met all of the following criteria were included in the study: male or female sex, Japanese pediatric patients aged <18 years; hyperammonemia (blood ammonia concentration ≥66 μg/dL); a coma grade of I or II^7^^–^^12^ or a diagnosis of suspected HE requiring medical treatment due to cryptogenic liver cirrhosis, biliary atresia, portal hypertension (including extrahepatic portal vein obstruction), congenital portosystemic shunt, Wilson’s disease, urea cycle disorders (chronic phase), or lysinuric protein intolerance as the underlying disease.

Patients with any of the following were excluded from the study: concurrent disease or medical history that might have affected the study assessment, including acute hepatitis or an acute exacerbation of chronic hepatitis with ascites requiring regular drainage, hemorrhage owing to rupture of esophageal or gastric varices, spontaneous bacterial peritonitis, or severe electrolyte abnormality as a concurrent disease; laboratory values at enrollment of blood urea nitrogen ≥25 mg/dL and serum creatinine ≥2.0 mg/dL, hemoglobin ≤7 g/dL, or serum potassium ≤2.5 mEq/L; the use of an antibacterial agent (excluding a dermatologic preparation) ≤6 days prior to the assessment of blood ammonia concentration, coma grade and number connection test (NCT)-A and test-B results, or ≤1 week prior to the commencement of the study medication; a history of allergy to rifamycin antibiotics; and being of reproductive age with the possibility of pregnancy, a willingness to become pregnant, or lactation.

### Endpoints

The primary efficacy endpoint was the blood ammonia concentration. The other efficacy endpoints were the changes in coma grade (Table 1) and psychoneurological function [assessed using NCT-A (time limit 120 sec) and NCT-B (180 sec)].^13^^,^^14^ NCT-A and NCT-B were administered to evaluable patients only. AEs, clinical laboratory test data, 12–lead electrocardiography data, the composition of the fecal microbiota, and vital signs were assessed as safety endpoints. The plasma concentration of rifaximin and pharmacokinetic parameters were also assessed.

**TABLE 1:** Mental state of hepatic encephalopathy (coma grade) in children and adults^7^^–^^12^

Coma grade	Children (5th Pediatric Hepatology Workshop)^a^		Adults (Inuyama Symposium)^b^	
	Older children	Infants	Psychiatric symptoms	Notes
I	• Not clearly conscious	• Laughs but does not laugh aloud when cradled • Does not laugh aloud but looks the adult in the eyes when cradled • Unable to look their parent in the eyes^c^	• Reversed sleep–wake rhythm • Euphoric, but sometimes depressed • Negligent attitude and uninterested	• Often only determined retrospectively
II	• Disoriented • Unable to say own name or date of birth • Exercises as intended (eg, grasping and releasing the right hand) • Speaks words, but often incorrectly	• Unable to look at their parent in the eyes^c^ • Tries to drink when a drink is shown or willing to suck a nipple when it is shown • Opens eyes and looks when called	• Disoriented (time, place) and identifies things wrongly • Abnormal behavior (eg, throwing money, disposing of cosmetics) • Sometimes drowsy (able to open eyes and speak in response to a normal call) • Sometimes behaves rudely, but willing to follow the physician’s instructions	• Not excited • No urinary or fecal incontinence • Flapping tremor
III	• Responds to simple commands (eg, handshake) • Barely opens eyes when called repeatedly	• Barely opens eyes when called repeatedly	• Defiant behavior, often accompanied by agitation or delirium • Lethargy (almost asleep) • Able to open eyes in response to external stimuli, but does not or cannot follow the physician’s instructions (able to respond to simple commands)	• Flapping tremor (when patient is cooperative) • Severely disoriented
IV	• Does not wake up in response to painful stimuli, but frowns or tries to brush these away		• Coma (complete loss of consciousness) • Responds to painful stimuli	• Brushes off and/or frowns in response to stimuli
V	• Does not respond to painful stimuli		• Deep coma • Does not respond to painful stimuli	_—_

^a^ The mental state of patients with hepatic encephalopathy was evaluated using the coma grade proposed by the Inuyama Symposium or the 5th Pediatric Hepatology Workshop.^5^^,^^7^^,^^8^^,^^11^^,^^12^ The 5th Pediatric Hepatology Workshop developed the criteria (coma grade), which are based on the evaluation of the level of consciousness of infants and young children, because it is often difficult to determine whether a child has impaired consciousness or not.

^b^ Physicians evaluated coma grade in adults according to the criteria proposed by the Inuyama Symposium.^5^^,^^7^^,^^11^^,^^12^

^c^ It is classified as grade I until 3 months of age and grade II after 3 months of age.

On day 1, the patients were hospitalized to undergo testing and investigation, and then they visited the study site or communicated by telephone or email for evaluations on days 4, 8, 12, 15, 29, 43, 57, and 71. In addition to these contacts, the status of the medication and AEs were assessed by telephone or email on days 22, 36, 50, 64, and 78. On day 85 or at the time of discontinuation of the study, end-of-treatment testing was performed.

### Analysis methods

Using the change in blood ammonia concentration over time as the primary efficacy endpoint, a sample size of 20 patients was selected on the basis of feasibility and the results of consultations with the participating medical institutions.

The efficacy of the treatment was analyzed using a full analysis set, which included all the enrolled patients, except for those who had not been administered rifaximin or evaluated with respect to drug efficacy on day 15. The safety of the treatment was assessed using a safety analysis set, comprising all the patients treated with rifaximin. The pharmacokinetic analysis was performed for patients who provided blood samples after day 8 during the treatment period.

For the efficacy of treatment assessment, the change from baseline at each time point and 95% confidence intervals (CIs) were calculated. The incidences of AEs, serious AEs (SAEs), and their severity were calculated. The composition of the fecal microbiota in samples was assessed through the next-generation sequencing of bacterial 16S rRNA at each time point; a diagram of the composition of each sample was created with respect to phylum, class, order, family, and genus; and qualitative changes were recorded.

In this study, CIs that did not include zero were accepted as indicating statistical significance. No missing, excluded, or abnormal data were imputed. Statistical analyses were performed using SAS version 9.3 or higher (SAS Institute Inc., Cary, NC, USA).

## RESULTS

### Demographics of the patients at baseline and their disease characteristics

The study was performed from January 2020 to October 2022 at 26 study sites in Japan. A total of 21 patients were enrolled, of whom 20 were included in the full analysis set, because the efficacy of the treatment was not evaluated in 1 patient (Supplemental Figure S1, http://links.lww.com/HC9/C387). All 21 patients were included in the safety and pharmacokinetic analyses. An important protocol deviation “related to prohibited concomitant medications” occurred in 1 (4.8%) patient. Eighteen (85.7%) patients completed the study. Three patients (14.3%) were withdrawn from the study for one of the following reasons: an AE that made continuation of the study difficult as determined by the investigator; a request by parents or legal guardian and the patient to withdraw from the study because going to school made it impossible to participate in this study; and, complication with pruritus that required the concomitant use of prohibited medication.

The median (Q1–Q3) duration of treatment with rifaximin was 84.0 (83.0–85.0) days. Treatment compliance was 98.5% (97.7%–99.2%). One patient experienced a moderate ADR related to the gastrointestinal tract, and the treatment regimen was modified as described above.

The age of the patients ranged from 5 to 16 years, and they had a median (Q1–Q3) age of 11.0 (8.0–14.0) years (Table 2). The most common underlying disease was congenital absence of the portal vein (8; 38.1%), followed by portal hypertension (7; 33.3%), liver cirrhosis (5; 23.8%), biliary atresia (4; 19.0%), and lysinuric protein intolerance (1; 4.8%). The mental state of HE was classified as “suspected HE” in 13 (61.9%) patients and coma grade I in 8 (38.1%).

**TABLE 2:** Demographic and other baseline characteristics

Parameter	Summary statistics
Number of patients in the safety analysis set	21
Female	14 (66.7)
Age, y, median (Q1–Q3)	11.0 (8.0–14.0)
5–11 y	11 (52.4)
12–17 y	10 (47.6)
Weight, kg, median (Q1–Q3)	43.80 (29.70–52.00)
Medical history and complications^a^	21 (100.0)
Previous medication	20 (95.2)
Underlying disease^b^	
Liver cirrhosis	5 (23.8)
Biliary atresia	4 (19.0)
Portal hypertension	7 (33.3)
Congenital absence of the portal vein (including congenital portosystemic shunt)	8 (38.1)
Lysinuric protein intolerance	1 (4.8)
Abnormal findings on standard 12–lead ECG	
No	10 (47.6)
Yes, not clinically significant	11 (52.4)
Yes, clinically significant	0 (0.0)
Blood ammonia concentration, μg/dL, median (Q1–Q3)	84.00 (76.64–105.00)
Mental state of HE	
Suspected HE	13 (61.9)
I	8 (38.1)
II	0 (0.0)
Number connection test–A result, sec, median (Q1–Q3)	45.4 (35.7–71.1)
Number connection test–A grade, median (Q1–Q3)	1.0 (1.0–2.0)
Number connection test–B result, sec, median (Q1–Q3)	84.3 (61.0–139.8)
Number of patients in the full analysis set	20
Duration of treatment, days, median (Q1–Q3)	84.0 (83.0–85.0)
Number of patients taking rifaximin	
Day 1–day 7	20 (100.0)
Day 8–day 14	20 (100.0)
Day 15–day 28	19 (95.0)
Day 29–day 56	19 (95.0)
Day 57–day 84	18 (90.0)
Day 85–day 91	7 (35.0)
Total	20 (100.0)
Final compliance, %, median (Q1–Q3)	98.5 (97.7–99.2)

*Note:* Data are presented as n (%) of patients unless otherwise indicated.

^a^ These items were collected and summarized together in the original dataset.

^b^ Multiple diseases were possible.

Abbreviations: ECG, electrocardiogram; HE, hepatic encephalopathy.

### Efficacy endpoints

The primary endpoint, blood ammonia concentration, was lower than at baseline at all subsequent visits, although the differences were not significant. The time-course diagram shows that the blood ammonia concentration was lower at week 4 [76.00 (62.00–110.00) μg/dL] than at baseline [median (Q1–Q3), 83.00 (74.93–112.11) μg/dL] and subsequently distributed on the lower side [week 8, 91.00 (62.00–113.00); week 12, 91.00 (69.00–109.00); end of treatment, 86.85 (63.00–103.00) μg/dL] (Figure 1). The changes in the blood ammonia concentration between baseline and weeks 2 and 12 were −13.66 (95% CI, −32.24 to 4.93) µg/dL and −10.30 (−30.22 to 9.62) µg/dL, respectively (Table 3). The change between baseline and the final evaluation was −10.95 (−27.64 to 5.73) µg/dL.

**FIGURE 1:**
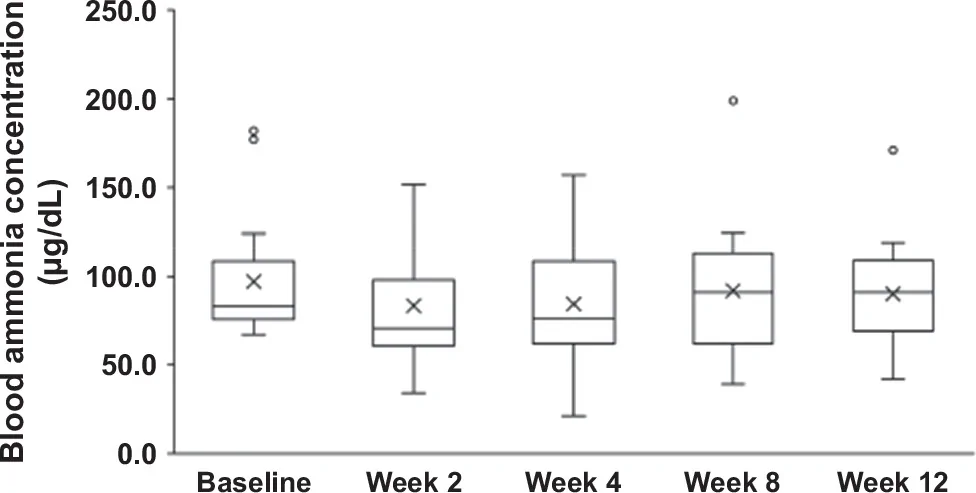
Changes in blood ammonia concentrations (full analysis set). × represents the mean; the box indicates the first quartile, median, and third quartile; the whisker indicates the maximum and minimum values; and ○ represents an outlier.

**TABLE 3:** Confidence intervals for the changes in the efficacy data

Item	Visit	N	Change	95% CI	
				Lower	Upper
Blood ammonia concentration, μg/dL	Visit 4 (week 2)	20	−13.66	−32.24	4.93
	Visit 5 (week 4)	19	−13.67	−31.11	3.78
	Visit 6 (week 8)	17	−6.62	−27.92	14.69
	Visit 7 (week 12)	17	−10.30	−30.22	9.62
	End of treatment	20	−10.95	−27.64	5.73
Mental state of HE	Visit 4 (week 2)	20	−0.18	−0.33	−0.02
	Visit 5 (week 4)	18	−0.17	−0.31	−0.02
	Visit 6 (week 8)	18	−0.22	−0.40	−0.05
	Visit 7 (week 12)	17	−0.24	−0.42	−0.05
	End of treatment	20	−0.25	−0.43	−0.07
Number connection test–A result, sec	Visit 4 (week 2)	19	−5.65	−13.77	2.47
	Visit 5 (week 4)	19	−4.22	−12.33	3.89
	Visit 6 (week 8)	18	−5.75	−14.32	2.82
	Visit 7 (week 12)	17	−3.76	−13.30	5.78
	End of treatment	19	−4.34	−12.97	4.29
Number connection test–A grade	Visit 4 (week 2)	19	−0.3	−0.7	0.2
	Visit 5 (week 4)	19	0.1	−0.4	0.5
	Visit 6 (week 8)	18	−0.3	−0.8	0.1
	Visit 7 (week 12)	17	0.0	−0.5	0.5
	End of treatment	19	−0.1	−0.5	0.4
Number connection test–B result, sec	Visit 4 (week 2)	19	−14.76	−26.58	−2.94
	Visit 5 (week 4)	19	−8.16	−23.13	6.82
	Visit 6 (week 8)	18	−13.67	−28.25	0.92
	Visit 7 (week 12)	17	−18.40	−31.86	−4.94
	End of treatment	19	−21.89	−35.35	−8.44

Abbreviations: CI, confidence interval; HE, hepatic encephalopathy.

With respect to the other endpoints, the changes in the mental state associated with HE from baseline were −0.18 (95% CI, −0.33 to −0.02) to week 2 and −0.24 (−0.42 to −0.05) to week 12. Between baseline and the final evaluation, the change was −0.25 (−0.43 to −0.07). These changes from baseline were all statistically significant (Table 3 and Supplemental Table S1, http://links.lww.com/HC9/C387).

There were young pediatric patients who were unable to take the test (Supplemental Table S2, http://links.lww.com/HC9/C387). Testing rate and completion rate for the NCT-B seemed to be lower than those for the NCT-A. Reasons for missing data were: patients were too young to take the test; patients had difficulty taking the test because they had autism; patients withdrew from the study and did not take the test; and patients did not follow the schedule and did not take the test. The results of the NCT-B showed significant decreases from baseline [−14.76 (95% CI, −26.58 to −2.94) seconds at week 2, −18.40 (−31.86 to −4.94) seconds at week 12, and −21.89 (−35.35 to −8.44) seconds at the final assessment].

### Safety assessments

A total of 24 AEs occurred in 11 (52.4%) patients (Supplemental Table S3, http://links.lww.com/HC9/C387). The most common AE was pyrexia [3 patients (14.3%)], followed by nasopharyngitis, diarrhea, and nausea [2 patients (9.5%) each], and another event [1 patient (4.8%)]. One patient experienced a single SAE (moderate enterocolitis), which was considered to be unrelated to the rifaximin administration. The patient withdrew from the study because of this SAE, which resolved later.

Three ADRs occurred in 3 (14.3%) patients [abdominal pain, nausea, and an increase in the circulating bilirubin concentration: 1 patient (4.8%) each]. For 1 patient who experienced moderate nausea, the dose was reduced.

No severe AEs occurred (Supplemental Table S4, http://links.lww.com/HC9/C387), and there were no deaths during the study. In addition, no clinically significant changes or abnormalities occurred in the laboratory parameters, vital signs, body mass, or electrocardiography parameters throughout the study.

The examination of the gut microbiota showed that bacteria belonging to the phyla *Firmicutes* and *Actinobacteria* predominated, but the proportions of these bacterial taxa varied among the patients. There were no marked changes in the composition of the gut microbiota between baseline and week 2 or week 12, and there were no consistent trends in the intra-patient or inter-patient variability.

### Pharmacokinetics

A population pharmacokinetic analysis was performed using 103 measurements of the plasma rifaximin concentration obtained from 21 patients, using a one-compartment model with first-order absorption. Zero, 5, and 11 hours after the first 400 mg dose of rifaximin of the day, the estimated steady-state pharmacokinetic parameters, calculated using the final model, were the same as those of the adult patients (Supplemental Table S5, http://links.lww.com/HC9/C387). Body weight was selected as a covariate for the clearance of the drug from the plasma and volume of distribution.

## DISCUSSION

To the best of our knowledge, this is the first clinical study in which the administration of rifaximin has been evaluated in pediatric patients. Overall, the present results demonstrate that rifaximin appears to improve mental state and psychoneurological function, although statistical significance was not obtained for the blood ammonia concentration. Regarding the safety, we found no new safety concerns or changes in the gut microbiota composition.

Although the results obtained were very similar to those for adults, we identified some differences in the baseline clinical characteristics of the children studied. Major differences in HE disease characteristics and patient backgrounds between pediatric and adult patients are summarized in Supplemental Table S6, http://links.lww.com/HC9/C387. For example, 98.8% of the adult patients in the rifaximin group had cirrhosis, with the principal underlying causes being hepatitis B, hepatitis C, and alcoholic cirrhosis;^5^ whereas congenital diseases, such as a congenital portosystemic shunt and lysinuric protein intolerance, had typically been diagnosed in the children. In addition, two-thirds of the present pediatric patients had suspected HE, whereas the previous study involved only adults with clinically confirmed HE. Another difference was the blood ammonia concentrations, which were lower in the pediatric patients than in the adults. Hyperammonemia appears to affect neurodevelopment, especially in children,^2^ and therefore their blood ammonia concentrations are typically more carefully controlled through dietary manipulations than those of adults.^4^^,^^15^ This difference might at least in part explain the differences between the pediatric and adult patients. Taken together with the present and previous results, it indicates that the present results are most applicable to pediatric patients with non-cirrhotic, shunt-related HE, as they were the majority of pediatric patients in this study.

Despite these differences, the efficacy findings for the pediatric patients were similar to those reported previously for the adult patients. The blood ammonia concentrations of the pediatric patients had decreased from their baseline levels by 15 days after the start of the treatment and remained low until the end of the study, representing a trend similar to that in the adult patients.^5^ The efficacy of rifaximin with respect to the amelioration of hyperammonemia in pediatric patients is also supported by the changes in the blood ammonia concentrations from baseline being similar to those demonstrated in adult patients (Supplemental Table S7, http://links.lww.com/HC9/C387). Thus, the hyperammonemia of children with HE would be expected to be reduced by rifaximin treatment.

The scores for the mental state of the pediatric patients showed significant decreases from baseline at all the time points assessed, consistent with the trends identified in the adult patients. Decreases in the NCT-A and NCT-B scores from baseline were also obtained until the last assessment. It should be noted that the learning effect has been suggested by repeated administration of NCTs.^16^ We implemented measures such as presenting numbers of each session randomly every time to reduce the learning effect of repetition; however, interpretation of the results should take into account that we cannot completely eliminate the learning effect. In the adult patients, the NCT-A and NCT-B scores decreased between baseline and 15 days after the start of the treatment and remained low until day 85. These improvements in the mental state and psychoneurological function appeared to be consistent with those of reductions in the blood ammonia concentrations. This study was a single-arm study and, therefore, the natural course of the disease, placebo effects, and regression toward the mean might have contributed to the clinical improvement. Considering such potential impacts, the efficacy of rifaximin has been evaluated using a combination of multiple assessments, namely, blood ammonia concentrations, coma grade, and NCTs, but not relying on a single efficacy endpoint. Overall, the present efficacy results were demonstrated primarily by clinical and neurocognitive measures rather than biochemical (ammonia) changes.

NCT reference values have not been established by age for Japanese pediatric patients. Therefore, the interpretation of the results is limited because some young pediatric patients were unable to take the tests. Indeed, we found that the NCT testing rate and completion rate in young pediatric patients tended to be low. In this study, the NCT was used as an indicator of change over time in individual patients rather than to compare the results between age groups. The present results demonstrated that the NCT-B value was significantly decreased from the baseline value, whereas no changes were found for the NCT-A value. NCT-A is a relatively simple task in which patients sequentially connect numbers. On the other hand, in the NCT-B, patients need to connect numbers and letters in an alternating sequence. Therefore, patients need a higher level of attention control and executive functions for the NCT-B than the NCT-A. Such differences in the test characteristics might have contributed to the differential results between the NCT-A and NCT-B. However, improvements in neurocognitive testing should be interpreted cautiously, given potential learning effects and lack of age-adjusted normative data because of some young pediatric patients who were unable to take the tests. It should also be noted that the differential degree of improvement between NCT-A and NCT-B could be explained by a combination of learning effects and a ceiling effect resulting from the relatively simple nature of NCT-A.

The results of the safety assessment were similar to those previously reported for adults. Despite the pediatric patients being treated with the same dose of rifaximin (1200 mg/day) as the adult patients, no SAEs occurred, and there were no AEs identified in the children that had not been identified in the adults. Although the sample size was small (n=4), rifaximin had no significant effects on the composition of the gut microbiota of the children, as for the adults.^17^ In addition, an analysis of the drug susceptibility of the isolated bacteria demonstrated that rifaximin was as effective against common bacterial strains as in the adult patients. Thus, overall, the results confirm the safety of the administration of rifaximin to children.

The pharmacokinetic data obtained for the pediatric patients were similar to those previously obtained for adults. The pharmacokinetic parameters were within the ranges or equivalent to those obtained for adult patients with HE, suggesting that the absorption of rifaximin in the gastrointestinal tracts of children is minimal (Supplemental Table S5, http://links.lww.com/HC9/C387).

As discussed above, the diseases responsible for the development of hyperammonemia in children tend to be rare congenital diseases. Therefore, the present study provides invaluable data regarding this rare patient group. Rifaximin would also be expected to ameliorate HE-associated hyperammonemia in patients with Wilson’s disease or urea cycle disorders. However, due to a limited number of patients and differences in the underlying pathological mechanisms, more studies are needed to determine if that is the case or not.

The study had some limitations. First, the sample size was small because of the nature of the clinical condition. Second, patients <5 years of age could not be studied because of a lack of previous safety data. Third, it was a single-arm study without a placebo group, and therefore, the contribution of the natural course of the disease, placebo effects, and regression towards the mean to the clinical improvement cannot be completely ruled out. Fourth, we only included patients whose psychoneurological function testing and gut microbial composition could be evaluated. Fifth, blood ammonia concentration may be variable in children with HE, and does not necessarily correlate with clinical symptoms. Therefore, it is critical to evaluate the efficacy of the study drug using multiple clinical indices in clinical studies. Sixth, NCT reference values have not been established by age for Japanese pediatric patients. Therefore, interpretation of the NCT results is limited, especially in young pediatric patients. Seventh, the microbiome analysis included only 4 patients who were observed for 12 weeks. Due to the limited sample size and the short duration of the treatment, subtle changes in the microbiome and the long-term efficacy of rifaximin have not been fully evaluated. Especially, it should be noted that the long-term impact of rifaximin in children with developing gut flora is not evaluated in this study. Therefore, it is not possible to draw a concrete conclusion about the impact of rifaximin on the microbiome in this study. To overcome these limitations, not only long-term monitoring of patients in real-world clinical settings, but also the development of age-appropriate neurocognitive tools for assessing neuropsychological functions, as well as collaborative multicenter pediatric studies, are needed.

In conclusion, rifaximin has been used to treat adult patients with HE for a long time, and it has a proven track record. The present results indicate that efficacy, safety, and pharmacokinetic profiles of rifaximin in children are consistent with those in adults.

## Supplementary Material
