# Supplementary material for: Efficacy, safety, and pharmacokinetics of rifaximin in pediatric patients with hepatic encephalopathy in Japan: An open-label study
Source: Hepatol Commun. 2026 Jul 15;10(8):e01002. doi: 10.1097/HC9.0000000000001002 (PMC13671586; doi:10.1097/HC9.0000000000001002)
Supplement: Supplementary file 1 [file hc9-10-e01002-s001.docx]

**Supplemental Digital Content**

**Efficacy, safety, and pharmacokinetics of rifaximin in pediatric patients with hepatic encephalopathy in Japan: an open-label study**

Hideaki Okajima, M.D., Ph.D. et al

**SDC, TABLE 1 -** Summary statistics for efficacy variables in the pediatric study

| Item | Visit | N | Mean | SD | Min | Q1 | Median | Q3 | Max |
| --- | --- | --- | --- | --- | --- | --- | --- | --- | --- |
| Coma grade | Baseline | 20 | 0.70 | 0.25 | 0.5 | 0.50 | 0.50 | 1.00 | 1.0 |
|  | Visit 4 (Week 2) | 20 | 0.53 | 0.26 | 0.0 | 0.50 | 0.50 | 0.50 | 1.0 |
|  | Visit 5 (Week 4) | 18 | 0.53 | 0.27 | 0.0 | 0.50 | 0.50 | 0.50 | 1.0 |
|  | Visit 6 (Week 8) | 18 | 0.47 | 0.32 | 0.0 | 0.50 | 0.50 | 0.50 | 1.0 |
|  | Visit 7 (Week 12) | 17 | 0.44 | 0.30 | 0.0 | 0.50 | 0.50 | 0.50 | 1.0 |
|  | End of treatment | 20 | 0.45 | 0.32 | 0.0 | 0.25 | 0.50 | 0.50 | 1.0 |
| Number connection test−A result, sec | Baseline | 20 | 60.23 | 35.61 | 23.9 | 33.80 | 44.70 | 91.30 | 120.0 |
|  | Visit 4 (Week 2) | 19 | 55.44 | 39.76 | 17.2 | 23.60 | 33.20 | 80.00 | 120.0 |
|  | Visit 5 (Week 4) | 19 | 57.30 | 36.37 | 16.8 | 26.60 | 50.90 | 65.40 | 120.0 |
|  | Visit 6 (Week 8) | 18 | 52.92 | 36.03 | 17.2 | 28.20 | 39.85 | 76.20 | 120.0 |
|  | Visit 7 (Week 12) | 17 | 56.24 | 38.94 | 15.0 | 24.30 | 41.40 | 72.50 | 120.0 |
|  | End of treatment | 19 | 52.74 | 38.18 | 15.0 | 22.30 | 37.20 | 72.50 | 120.0 |
| Number connection test−B result, sec | Baseline | 20 | 102.26 | 52.86 | 32.9 | 57.65 | 83.35 | 150.40 | 180.0 |
|  | Visit 4 (Week 2) | 19 | 89.67 | 54.60 | 27.0 | 48.30 | 71.30 | 119.90 | 180.0 |
|  | Visit 5 (Week 4) | 19 | 93.69 | 59.53 | 27.5 | 45.50 | 77.00 | 180.00 | 180.0 |
|  | Visit 6 (Week 8) | 18 | 83.84 | 53.18 | 19.8 | 43.70 | 65.50 | 116.70 | 180.0 |
|  | Visit 7 (Week 12) | 17 | 80.00 | 55.78 | 19.1 | 42.80 | 55.80 | 109.50 | 180.0 |
|  | End of treatment | 19 | 76.27 | 53.86 | 19.1 | 35.10 | 54.30 | 109.50 | 180.0 |

Q, quartile.

**SDC, TABLE 2 – Status of executing number connection test-A and -B by age**

| Age | No. of patients in the full analysis set | | No. of patients who took test | No. of tests patients took | Testing rate (%) | No. of completed tests | Completion rate (%) | Number of missed tests |
| --- | --- | --- | --- | --- | --- | --- | --- | --- |
| Number connection test-A | | | | | | | | |
| 5 | 1 | | 1 | 1 | 20 | 0 | 0 | 4^a^ |
| 6 | 1 | | 1 | 5 | 100 | 0 | 0 | 0 |
| 7 | 2 | | 1 | 5 | 50 | 4 | 80 | 5^b^ |
| 8 | 3 | | 3 | 12 | 80 | 12 | 100 | 3^c^ |
| 9 | 0 | | - | - | - | - | - | - |
| 10 | 1 | | 1 | 5 | 100 | 5 | 100 | 0 |
| 11 | 3 | | 3 | 14 | 93 | 14 | 100 | 1^d^ |
| 12 | 1 | | 1 | 1 | 20 | 1 | 100 | 4^c^ |
| 13 | 2 | | 2 | 10 | 100 | 10 | 100 | 0 |
| 14 | 2 | | 2 | 7 | 70 | 7 | 100 | 3^c^ |
| 15 | 2 | | 2 | 10 | 100 | 10 | 100 | 0 |
| 16 | 3 | | 3 | 15 | 100 | 15 | 100 | 0 |
| 17 | 0 | | - | - | - | - | - | - |
| Number connection test-B | | | | | | | | |
| 5 | | 1 | 0 | 0 | 0 | 0 | 0 | 5^a^ |
| 6 | | 1 | 1 | 5 | 100 | 0 | 0 | 0 |
| 7 | | 2 | 1 | 5 | 50 | 5 | 100 | 5^b^ |
| 8 | | 3 | 3 | 12 | 80 | 10 | 83 | 3^c^ |
| 9 | | 0 | - | - | - | - | - | - |
| 10 | | 1 | 1 | 5 | 100 | 5 | 100 | 0 |
| 11 | | 3 | 3 | 14 | 93 | 14 | 100 | 1^d^ |
| 12 | | 1 | 1 | 1 | 20 | 1 | 100 | 4^c^ |
| 13 | | 2 | 2 | 10 | 100 | 9 | 90 | 0 |
| 14 | | 2 | 2 | 7 | 70 | 7 | 100 | 3^c^ |
| 15 | | 2 | 2 | 10 | 100 | 10 | 100 | 0 |
| 16 | | 3 | 3 | 15 | 100 | 15 | 100 | 0 |
| 17 | | 0 | - | - | - | - | - | - |

^a^Patient was too young to take the test.

^b^Patient had difficulty taking the test because the patient had autism.

^c^Patient withdrew from the study and did not take the test.

^d^Patient did not follow the schedule and did not take the test.

**SDC, TABLE 3 -** Adverse events and adverse drug reactions in the pediatric study

| System/organ class  Preferred terms | Adverse events | | | Adverse drug reactions | | |
| --- | --- | --- | --- | --- | --- | --- |
|  | No. of events | No. of patients | Incidence  (%) | No. of events | No. of patients | Incidence  (%) |
| Total | 24 | 11 | 52.4 | 3 | 3 | 14.3 |
| Infections and infestations | 4 | 3 | 14.3 | _―_ | _―_ | _―_ |
| Hordeolum | 1 | 1 | 4.8 | _―_ | _―_ | _―_ |
| Nasopharyngitis | 3 | 2 | 9.5 | _―_ | _―_ | _―_ |
| Nervous system disorders | 1 | 1 | 4.8 | _―_ | _―_ | _―_ |
| Headache | 1 | 1 | 4.8 | _―_ | _―_ | _―_ |
| Respiratory, thoracic, and mediastinal disorders | 2 | 2 | 9.5 | _―_ | _―_ | _―_ |
| Epistaxis | 1 | 1 | 4.8 | _―_ | _―_ | _―_ |
| Rhinitis allergic | 1 | 1 | 4.8 | _―_ | _―_ | _―_ |
| Gastrointestinal disorders | 8 | 6 | 28.6 | 2 | 2 | 9.5 |
| Abdominal pain | 1 | 1 | 4.8 | 1 | 1 | 4.8 |
| Diarrhea | 3 | 2 | 9.5 | _―_ | _―_ | _―_ |
| Enterocolitis | 1 | 1 | 4.8 | _―_ | _―_ | _―_ |
| Nausea | 2 | 2 | 9.5 | 1 | 1 | 4.8 |
| Vomiting | 1 | 1 | 4.8 | _―_ | _―_ | _―_ |
| Skin and subcutaneous tissue disorders | 2 | 1 | 4.8 | _―_ | _―_ | _―_ |
| Erythema | 1 | 1 | 4.8 | _―_ | _―_ | _―_ |
| Pruritus | 1 | 1 | 4.8 | _―_ | _―_ | _―_ |
| General disorders and administration site conditions | 5 | 5 | 23.8 | _―_ | _―_ | _―_ |
| Influenza−like illness | 1 | 1 | 4.8 | _―_ | _―_ | _―_ |
| Peripheral edema | 1 | 1 | 4.8 | _―_ | _―_ | _―_ |
| Pyrexia | 3 | 3 | 14.3 | _―_ | _―_ | _―_ |
| Laboratory data | 1 | 1 | 4.8 | 1 | 1 | 4.8 |
| High circulating bilirubin concentration | 1 | 1 | 4.8 | 1 | 1 | 4.8 |
| Injury, poisoning, and complications of procedures | 1 | 1 | 4.8 | _―_ | _―_ | _―_ |
| Second−degree burns | 1 | 1 | 4.8 | _―_ | _―_ | _―_ |

**SDC, TABLE 4 -** Adverse events and adverse drug reactions, classified by severity in the pediatric study

| System/organ class  Preferred terms | Adverse events | | | | | | Adverse drug reactions | | | | | |
| --- | --- | --- | --- | --- | --- | --- | --- | --- | --- | --- | --- | --- |
|  | Mild | | Moderate | | Severe | | Mild | | Moderate | | Severe | |
|  | No. of patients | Incidence  (%) | No. of patients | Incidence  (%) | No. of patients | Incidence  (%) | No. of patients | Incidence  (%) | No. of patients | Incidence  (%) | No. of patients | Incidence  (%) |
| Total | 10 | 47.6 | 3 | 14.3 | 0 | 0.0 | 2 | 9.5 | 1 | 4.8 | 0 | 0.0 |
| Infections and infestations | 3 | 14.3 | 0 | 0.0 | 0 | 0.0 | _―_ | _―_ | _―_ | _―_ | _―_ | _―_ |
| Hordeolum | 1 | 4.8 | 0 | 0.0 | 0 | 0.0 | _―_ | _―_ | _―_ | _―_ | _―_ | _―_ |
| Nasopharyngitis | 2 | 9.5 | 0 | 0.0 | 0 | 0.0 | _―_ | _―_ | _―_ | _―_ | _―_ | _―_ |
| Nervous system disorders | 1 | 4.8 | 0 | 0.0 | 0 | 0.0 | _―_ | _―_ | _―_ | _―_ | _―_ | _―_ |
| Headache | 1 | 4.8 | 0 | 0.0 | 0 | 0.0 | _―_ | _―_ | _―_ | _―_ | _―_ | _―_ |
| Respiratory, thoracic, and mediastinal disorders | 2 | 9.5 | 0 | 0.0 | 0 | 0.0 | _―_ | _―_ | _―_ | _―_ | _―_ | _―_ |
| Epistaxis | 1 | 4.8 | 0 | 0.0 | 0 | 0.0 | _―_ | _―_ | _―_ | _―_ | _―_ | _―_ |
| Rhinitis allergic | 1 | 4.8 | 0 | 0.0 | 0 | 0.0 | _―_ | _―_ | _―_ | _―_ | _―_ | _―_ |
| Gastrointestinal disorders | 3 | 14.3 | 3 | 14.3 | 0 | 0.0 | 1 | 4.8 | 1 | 4.8 | 0 | 0.0 |
| Abdominal pain | 1 | 4.8 | 0 | 0.0 | 0 | 0.0 | 1 | 4.8 | 0 | 0.0 | 0 | 0.0 |
| Diarrhea | 2 | 9.5 | 0 | 0.0 | 0 | 0.0 | _―_ | _―_ | _―_ | _―_ | _―_ | _―_ |
| Enterocolitis | 0 | 0.0 | 1 | 4.8 | 0 | 0.0 | _―_ | _―_ | _―_ | _―_ | _―_ | _―_ |
| Nausea | 0 | 0.0 | 2 | 9.5 | 0 | 0.0 | 0 | 0.0 | 1 | 4.8 | 0 | 0.0 |
| Vomiting | 1 | 4.8 | 0 | 0.0 | 0 | 0.0 | _―_ | _―_ | _―_ | _―_ | _―_ | _―_ |
| Skin and subcutaneous tissue disorders | 1 | 4.8 | 0 | 0.0 | 0 | 0.0 | _―_ | _―_ | _―_ | _―_ | _―_ | _―_ |
| Erythema | 1 | 4.8 | 0 | 0.0 | 0 | 0.0 | _―_ | _―_ | _―_ | _―_ | _―_ | _―_ |
| Pruritus | 1 | 4.8 | 0 | 0.0 | 0 | 0.0 | _―_ | _―_ | _―_ | _―_ | _―_ | _―_ |
| General disorders and administration site conditions | 5 | 23.8 | 0 | 0.0 | 0 | 0.0 | _―_ | _―_ | _―_ | _―_ | _―_ | _―_ |
| Influenza−like illness | 1 | 4.8 | 0 | 0.0 | 0 | 0.0 | _―_ | _―_ | _―_ | _―_ | _―_ | _―_ |
| Peripheral edema | 1 | 4.8 | 0 | 0.0 | 0 | 0.0 | _―_ | _―_ | _―_ | _―_ | _―_ | _―_ |
| Pyrexia | 3 | 14.3 | 0 | 0.0 | 0 | 0.0 | _―_ | _―_ | _―_ | _―_ | _―_ | _―_ |
| Laboratory data | 1 | 4.8 | 0 | 0.0 | 0 | 0.0 | 1 | 4.8 | 0 | 0.0 | 0 | 0.0 |
| High circulating bilirubin concentration | 1 | 4.8 | 0 | 0.0 | 0 | 0.0 | 1 | 4.8 | 0 | 0.0 | 0 | 0.0 |
| Injury, poisoning, and procedural complications | 0 | 0.0 | 1 | 4.8 | 0 | 0.0 | _―_ | _―_ | _―_ | _―_ | _―_ | _―_ |
| Second−degree burns | 0 | 0.0 | 1 | 4.8 | 0 | 0.0 | _―_ | _―_ | _―_ | _―_ | _―_ | _―_ |

**SDC, TABLE 5 -** Population pharmacokinetic parameters for rifaximin at steady state after the first dose in patients with hepatic encephalopathy treated with 400 mg oral rifaximin three times daily after meals in the present pediatric study and a previous study of adults

| Participants | No. of patients | C_max_ (ng/mL) | T_max_ (h) | AUC_0–4 h_ (ng·h/mL) |
| --- | --- | --- | --- | --- |
| Children^a^ | 21 | 21.4 ± 9.5 | 2.0 [1.8, 2.0] | 75.7 ± 33.7 |
| Adults^b^ | 38 | 32.9 ± 20.6 | 2.0 [0.0, 4.0] | 88.1 ± 55.8 |

T_max_ is shown as the median [range].

^a^Estimated using the final population pharmacokinetic model in pediatric patients in the present study

^b^Calculated from actual measured values in the adult study^1^

C_max_, maximum drug concentration; T_max_, time to maximum concentration.

**SDC, TABLE 6 –** Summary of HE disease characteristics in pediatric and adult patients

|  | Pediatric patients | Adult patients^a, b^ |
| --- | --- | --- |
| Major underlying diseases | Congenital disorders and metabolic disorders, such as congenital portosystemic shunts and urea cycle disorders | Chronic liver diseases, primarily cirrhosis (viral cirrhosis, alcoholic cirrhosis, etc.) |
| Pathophysiology | Related to congenital abnormalities and metabolic disorders | Liver cirrhosis-related impaired liver functions and portosystemic shunt |
| Clinical symptoms | Varies with age and nonspecific | Wide spectrum of symptoms from subclinical to overt encephalopathy |
| Diagnostic features | Diagnosis is difficult because of diverse underlying diseases and developmental characteristics. Screening methods unique to pediatric patients have not been established. | Diagnosed based on the ammonia concentrations and comprehensive assessment by physician, while ruling out other functional brain disorders. |
| Population | Diverse backgrounds including rare diseases | Patients with liver cirrhosis |

^a^Forna L, Bozomitu L, Lupu VV, et al. Pediatric perspectives on liver cirrhosis: unravelling clinical patterns and therapeutic challenges. J Clin Med. 2024;13:4275.

^b^Bartlett JA, Kohli R. Hepatic encephalopathy in children. Indian J Pediatr. 2024;91:280–285.

HE, hepatic encephalopathy.

**SDC, TABLE 7 -** Changes in the blood ammonia concentration from baseline in pediatric patients in the present study and adult patients in the previous Japanese phase III study^a^

|  | Time point^b^ | Pediatric study^c^ | | Adult phase III study | |
| --- | --- | --- | --- | --- | --- |
|  |  | Mean ± SD | No. of patients | Mean ± SD | No. of patients |
| Blood ammonia concentration (μg/dL) | Baseline | 96.99 ± 33.40 | 20 | 134.47 ± 49.95 | 69 |
| Change from baseline (μg/dL) | Week 2 | −13.66 ± 39.71 | 20 | −12.25 ± 56.33 | 69 |
|  | Week 4 | −13.67 ± 36.19 | 19 | −19.03 ± 50.75 | 66 |
|  | Week 8 | −6.62 ± 41.43 | 17 | −31.21 ± 45.78 | 61 |
|  | Week 12 | −10.30 ± 38.74 | 17 | −28.99 ± 46.17 | 59 |
|  | End of treatment^d^ | −10.95 ± 35.64 | 20 | −22.53 ± 50.40 | 69 |

^a^Suzuki K, Endo R, Takikawa Y, et al. Efficacy and safety of rifaximin in Japanese patients with hepatic encephalopathy: A phase II/III, multicenter, randomized, evaluator−blinded, active−controlled trial and a phase III, multicenter, open trial. Hepatol Res. 2018;48:411–423.

^b^Pediatric study: baseline = Day 1, Week 2 = Days 14–18, Week 4 = Days 25–33, Week 8 = Days 54–61, Week 12 = Days 82–92; Adult phase III study: Week 4 = Days 22–36, Week 8 = Days 50–64, Week 12 = Days 77–91. Day of initiation of treatment in the pediatric study: Day 1.

^c^Present study of pediatric patients with HE.

^d^Last time point at which each patient was evaluated.

HE, hepatic encephalopathy.

**SDC, Materials and Methods**

Of the 84 patients who participated in the previous phase II/III multicenter, randomized, evaluator-blinded, active-controlled study that aimed to evaluate the efficacy and safety of the administration of rifaximin in adult Japanese patients with HE, 38 patients provided blood samples for pharmacokinetic analyses.^1^ A population pharmacokinetic analysis was performed using a one-compartment model, assuming first-order absorption.

**Reference**

1. Suzuki K, Endo R, Takikawa Y, et al. Efficacy and safety of rifaximin in Japanese patients with hepatic encephalopathy: A phase II/III, multicenter, randomized, evaluator-blinded, active-controlled trial and a phase III, multicenter, open trial. Hepatol Res. 2018;48:411–423.

Enrolled (21)

Drug administered (21)

Completed study (18)

Discontinued study (3)

Adverse events (1)

Other reasons (2)

**SDC, FIGURE 1:** Disposition of the patients.

STROBE Statement—checklist of items that should be included in reports of observational studies

|  | Item No | Recommendation | Page  No |
| --- | --- | --- | --- |
| **Title and abstract** | 1 | (*a*) Indicate the study’s design with a commonly used term in the title or the abstract | 3 |
|  |  | (*b*) Provide in the abstract an informative and balanced summary of what was done and what was found | 3 |
| Introduction | | | |
| Background/rationale | 2 | Explain the scientific background and rationale for the investigation being reported | 4 |
| Objectives | 3 | State specific objectives, including any prespecified hypotheses | 4 |
| Methods | | | |
| Study design | 4 | Present key elements of study design early in the paper | 4-5 |
| Setting | 5 | Describe the setting, locations, and relevant dates, including periods of recruitment, exposure, follow-up, and data collection | 4-5 |
| Participants | 6 | (*a*) *Cohort study*—Give the eligibility criteria, and the sources and methods of selection of participants. Describe methods of follow-up  *Case-control study*—Give the eligibility criteria, and the sources and methods of case ascertainment and control selection. Give the rationale for the choice of cases and controls  *Cross-sectional study*—Give the eligibility criteria, and the sources and methods of selection of participants | 6 |
|  |  | (*b*) *Cohort study*—For matched studies, give matching criteria and number of exposed and unexposed  *Case-control study*—For matched studies, give matching criteria and the number of controls per case | NA |
| Variables | 7 | Clearly define all outcomes, exposures, predictors, potential confounders, and effect modifiers. Give diagnostic criteria, if applicable | 6-7 |
| Data sources/ measurement | 8* | For each variable of interest, give sources of data and details of methods of assessment (measurement). Describe comparability of assessment methods if there is more than one group | 7 |
| Bias | 9 | Describe any efforts to address potential sources of bias | NA |
| Study size | 10 | Explain how the study size was arrived at | 7 |
| Quantitative variables | 11 | Explain how quantitative variables were handled in the analyses. If applicable, describe which groupings were chosen and why | NA |
| Statistical methods | 12 | (*a*) Describe all statistical methods, including those used to control for confounding | NA |
|  |  | (*b*) Describe any methods used to examine subgroups and interactions | NA |
|  |  | (*c*) Explain how missing data were addressed | 7 |
|  |  | (*d*) *Cohort study*—If applicable, explain how loss to follow-up was addressed  *Case-control study*—If applicable, explain how matching of cases and controls was addressed  *Cross-sectional study*—If applicable, describe analytical methods taking account of sampling strategy | NA |
|  |  | (*e*) Describe any sensitivity analyses | NA |
| Results | | | |
| Participants | 13* | (a) Report numbers of individuals at each stage of study—eg numbers potentially eligible, examined for eligibility, confirmed eligible, included in the study, completing follow-up, and analysed | 7-8 |
|  |  | (b) Give reasons for non-participation at each stage | 78 |
|  |  | (c) Consider use of a flow diagram | Suppl Fig 1 |
| Descriptive data | 14* | (a) Give characteristics of study participants (eg demographic, clinical, social) and information on exposures and potential confounders | 8, Table 2 |
|  |  | (b) Indicate number of participants with missing data for each variable of interest | NA |
|  |  | (c) *Cohort study*—Summarise follow-up time (eg, average and total amount) | NA |
| Outcome data | 15* | *Cohort study*—Report numbers of outcome events or summary measures over time | 8-9 |
|  |  | *Case-control study—*Report numbers in each exposure category, or summary measures of exposure | NA |
|  |  | *Cross-sectional study—*Report numbers of outcome events or summary measures | NA |
| Main results | 16 | (*a*) Give unadjusted estimates and, if applicable, confounder-adjusted estimates and their precision (eg, 95% confidence interval). Make clear which confounders were adjusted for and why they were included | 8-9, Suppl Tables 1-7, Fig 1, Table 3 |
|  |  | (*b*) Report category boundaries when continuous variables were categorized | NA |
|  |  | (*c*) If relevant, consider translating estimates of relative risk into absolute risk for a meaningful time period | NA |
| Other analyses | 17 | Report other analyses done—eg analyses of subgroups and interactions, and sensitivity analyses | NA |
| Discussion | | | |
| Key results | 18 | Summarise key results with reference to study objectives | 9-11 |
| Limitations | 19 | Discuss limitations of the study, taking into account sources of potential bias or imprecision. Discuss both direction and magnitude of any potential bias | 11-12 |
| Interpretation | 20 | Give a cautious overall interpretation of results considering objectives, limitations, multiplicity of analyses, results from similar studies, and other relevant evidence | 12 |
| Generalisability | 21 | Discuss the generalisability (external validity) of the study results | NA |
| Other information | | | |
| Funding | 22 | Give the source of funding and the role of the funders for the present study and, if applicable, for the original study on which the present article is based | 1 |

*Give information separately for cases and controls in case-control studies and, if applicable, for exposed and unexposed groups in cohort and cross-sectional studies.

**Note:** An Explanation and Elaboration article discusses each checklist item and gives methodological background and published examples of transparent reporting. The STROBE checklist is best used in conjunction with this article (freely available on the Web sites of PLoS Medicine at http://www.plosmedicine.org/, Annals of Internal Medicine at http://www.annals.org/, and Epidemiology at http://www.epidem.com/). Information on the STROBE Initiative is available at www.strobe-statement.org.
